# Surface Shape of the Calcaneal Tuberosity and the Occurrence of Retrocalcaneal Bursitis among Runners

**DOI:** 10.3390/ijerph18062860

**Published:** 2021-03-11

**Authors:** Agnieszka Wnuk-Scardaccione, Ewa Mizia, Klaudia Zawojska, Jan Bilski, Jakub Wojdyła

**Affiliations:** 1 Department of Biomechanics and Kinesiology, Faculty of Health Sciences, Jagiellonian University Collegium Medicum, 31-008 Krakow, Poland; klaudia.zawojska@uj.edu.pl (K.Z.); jan.bilski@uj.edu.pl (J.B.); 2 Department of Anatomy, Jagiellonian University Collegium Medicum, 31-008 Krakow, Poland; ewa.mizia@uj.edu.pl; 3 Faculty of Applied Mathematics, AGH University of Science and Technology, 30-059 Krakow, Poland; jwojdyla@agh.edu.pl

**Keywords:** retrocalcaneal bursa, calcaneal tuberosity, running

## Abstract

Purpose: The aim of the study was to establish the relationship between the shape of the calcaneal tuberosity (flat, stepped, rounded, normal) and the probability that retrocalcaneal bursitis among people who train running regularly. Methods: The study included a group of 30 runners who suffered from retrocalcaneal bursitis in the past, and 30 people who never had symptoms of this disease. The study was based on a diagnostic survey, as well as on clinical examination. The surface of the calcaneal tuberosity and the slope of the calcaneus were assessed using X-rays. The mobility of the bursa, its surface size, the thickness of the Achilles tendon and its attachment rate were established during an ultrasound examination. Results: Flat surface of the calcaneal tuberosity increases fourfold the risk of suffering from retrocalcaneal bursitis (OR = 4.3). The people whose calcaneus slope is above 25° are at increased risk of suffering from such an inflammation compared with the people whose calcaneus bone is more horizontal (OR = 2.8). The analysis shows that the thickness of the Achilles tendon (*p* = 0.001), the surface size of the bursa (*p* = 0.009), as well as the flat surface of the calcaneal tuberosity (*p* = 0.008) are strongly associated with the occurrence of retrocalcaneal bursitis. Conclusions: The flat shape of the calcaneal tuberosity increases the risk of bursitis. The risk of inflammation is higher when the Achilles tendon is thicker and the surface of the bursa is smaller than normal.

## 1. Introduction

Physical activity provides people with a variety of health benefits [1]. However, a risk of injury increases significantly when the training intensity, volume, and load are greater [2,3]. Running is one of the most popular sports disciplines in the world [4]. According to the latest studies from 2018, the ten longest 42 km and 195 m runs were completed by over 33 thousand people. In the Netherlands in 2013, 2.1 million people declared their participation in at least one street run [5].

This discipline is easily available. Ooms et al. [6] proved that 4–5 months after finishing a 6-week start-to-run program, 69% of the participants continued to be physically active through regular running workouts (152 min/a week). This discipline, then, has a great influence on the promotion of positive patterns of physical activity.

With the increasing popularity of running, the number of injuries that affect the people who have chosen this sports discipline has also risen (Running Related Injuries—RRI) [7,8]. The data on RRI vary, depending on the selected group, e.g., the mean incidence of RRI in the group of amateurs is 17.8 (95% CI 16.7–19.1) and in the group of professionals—7.7 (95% CI 6.9–8.7) for 1000 h of training [9]. According to the current research, a break from training is usually caused by knee or foot-related injury [10]. The analysis of the epidemiological studies shows that in the European Union population, on average, every minute one person gets injured due to motor activity [11]. EU Injury Database provided data showing that every year, about 5.2 million people have problems concerning the musculoskeletal system’s injuries, and 18% of them needed at least one-day hospitalization [12].

Based on histopathological examination of the inflamed retrocalcaneal bursa, the fibrocartilaginous walls show degenerative changes and/or a certain degree of calcification, together with the hypertrophy of the fibrous membrane and a build-up of fluid inside the bursa [13]. Epidemiological data are not easy to establish. Some information concerning retrocalcaneal bursitis is assigned to its more general name—Achilles tendinopathy. It must also be kept in mind that these problems can coexist. General inflammation in this area is a common ailment that affects not only the people who do sports, but also the ones who are not involved in any physical activity. It has been estimated that at least 24% of physically active people will have some issues with the Achilles tendon at least once in their lives [14]. The occurrence of inflammation in this area of the lower extremity constitutes from 6 to 17% of all injuries in people training running [15]. Additionally, the largest percentage concerns the people who take part in medium or long-distance running. In this group, from 7% to 9% of retrocalcaneal bursitis cases occur every year [16].

It is believed that the bursitis might be associated with a prominence of the posterosuperior angle of the calcaneus. There are three most common variations in the shape of the superior tuberosity of the calcaneus: normal, flat, and prominent. Although the prominent is known to be associated with bursitis, it is not uncommon to find retrocalcaneal bursitis in runners without any calcaneal deformity. Compression of the bursa between the calcaneus and the Achilles tendon occurs every time the ankle is dorsiflexed, especially in uphill running [17].

According to some sources, the co-occurrence of a bigger eminence of the calcaneal tuberosity and the inflammation of the retrocalcaneal bursa, related to its insertion into the bone, is 25% [18]. There are no reports of any anatomical variations in the surface shape of the calcaneal tuberosity, nor of any other possible associations with the occurrence of retrocalcaneal bursitis. The study’s primary aim was to inspect if also the flat shape of calcaneal tuberosity is associated with a higher risk of retrocalcaneal bursitis among runners.

## 2. Materials and Methods

### 2.1. Participants

The data collection lasted for 2 years. The Bioethics Committee of the Jagiellonian University granted permission for this research (permission number 122.6120.314.201), and the study was performed in accordance with the recommendation in the Declaration of Helsinki. 56 people were invited to participate in the research. All of them had sought medical attention at the Medical Centre REHABILITANCI.ORG in Kraków, due to retrocalcaneal bursitis and they had declared running as one of their sports disciplines (minimum 3 times a week, about 30 km/weekly).

A positive and informed consent for the participation in the research was given by 33 people. Three people, who at the time of the clinical evaluation complied with the exclusion criteria, were excluded from the group.

Inclusion criteria were as follows:

1. Pain in the retrocalcaneal bursa area with one of the two following findings,
  (a) Distinct tenderness of retrocalcaneal bursa with no pain in the neighboring structures,
  (b) Ultrasonographic changes defined as local inflammation,
2. Diffuse pain in the posterior region of the ankle with local tenderness of the retrocalcaneal bursa and ultrasonographic changes (as described above).

Factors for exclusion were as following:

1. Lack of patient’s consent to participate in the study,
2. Previous or current issues—neurological, orthopedic, or rheumatologic (fibromyalgia, rheumatoid arthritis, psoriatic arthritis, or other systemic diseases that occur with arthritis),
3. Cancer, congenital disorders, scoliosis, a limb length discrepancy,
4. Previous spinal injuries,
5. Operations conducted within the ankle joint and/or foot,
6. Degenerative changes in the hip, knee or subtalar joints occurring with the reduced mobility in these joints.
7. Hearing impairment.

Current and unilateral inflammation of the retrocalcaneal bursa was confirmed in every person, based on the clinical examination, symptoms, and an ultrasound scan.

**Figure 1:**
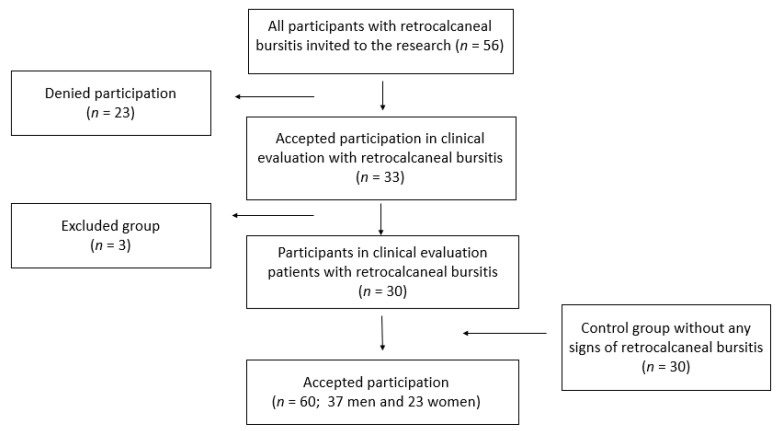
Flow-chart of participant recruitment.

Thirty people were examined and randomly assigned to the control group. All of them are recreational or professional runners, and their training meets the requirements of this study. The control group had to meet the same inclusion criteria as the test group. The invitation to participate in the research was issued to 56 people from different running groups and sports centers (Figure 1).

### 2.2. Experimental Protocol

The study was based on a diagnostic survey with the use of the author’s questionnaire, as well as on a clinical examination of the patient with the use of the following tools: an x-ray machine (Q-RAD DS of the Quantum Medical Imaging company, Bohemia, NY, USA) and an ultrasound machine (MyLab25Gold, produced by Esaote, Genoa, Italy).

For the participants’ clinical evaluation, a touch test was used, following the methodology of Deschamps et al. [19]. To confirm the diagnosis, every patient was subjected to an ultrasound examination, proving the presence of inflammation. At the same time, additional diseases connected with the place where the Achilles tendon attaches to the surface of the calcaneal tuberosity were excluded. If the patient met the inclusion criteria, the project manager offered them the opportunity to participate in the research. The proper testing was conducted after the inflammation within the retrocalcaneal bursa had subsided so that the pain present in that areas would not affect the results of the study.

In the questionnaire-based survey the respondents declared an average number of running workouts a week and an average weekly distance measured in kilometers. Some basic socio-demographic data were collected (age, sex, marital status, place of residence, type of professional activity). The respondents were asked about the details of the running training—was it the main form of the physical activity, how many hours a week did it take, and how many kilometers the participant ran weekly. Questions were also asked about the type of the running shoes, terrain, and the type of ground.

The additional questions concerned their history of injuries, which led to a considerably long break in sports training, as well as the self-assessment of their own health. Attention was paid to the type and location of any possible pain (other than related to retrocalcaneal bursitis) mentioned by the patient as it could influence the study results.

**Figure 2:**
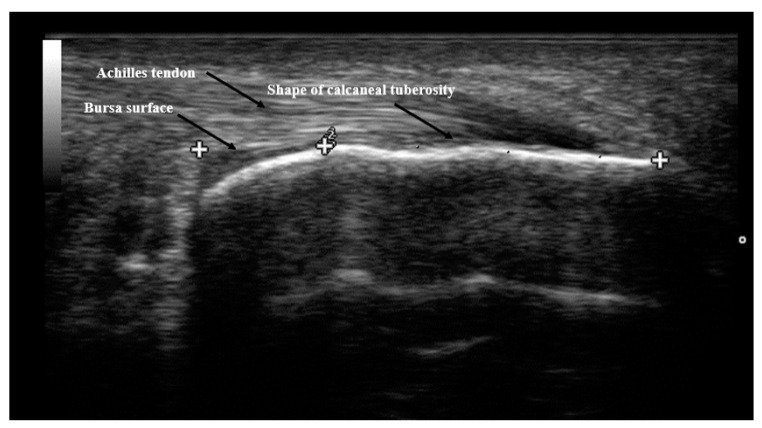
Ultrasonographic image of flat shape of the calcaneal tuberosity.

**Figure 3:**
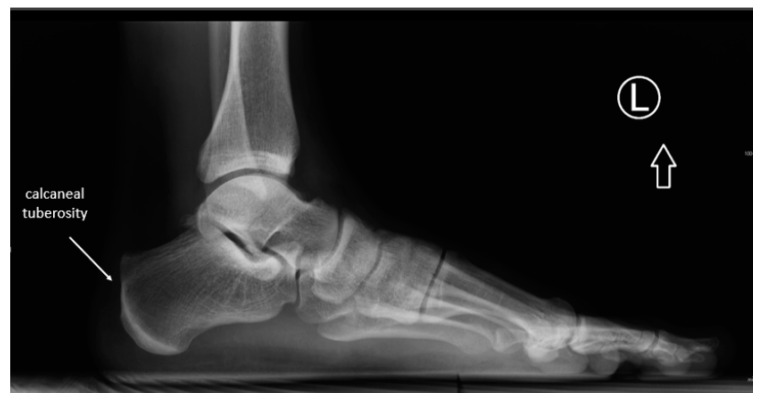
X-ray image of the flat shape of the calcaneal tuberosity.

**Figure 4:**
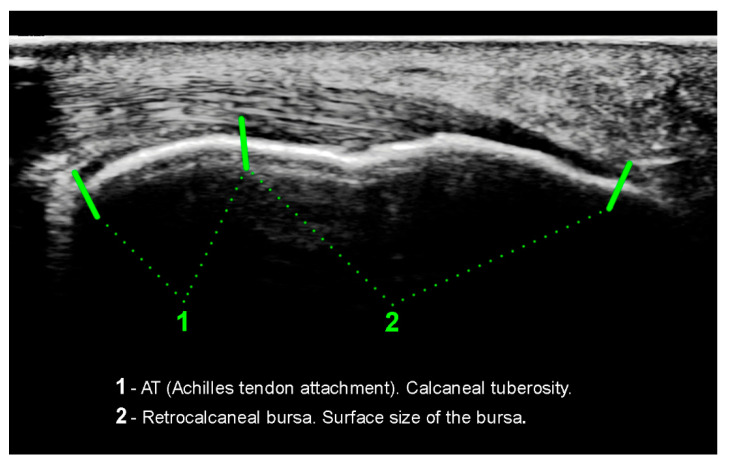
Ultrasonographic image presenting the bursa size measurement.

The senior author made the radiographic measurements based on his clinical examination and radiographic review. All patients had lateral view standing radiographs taken of the ankle. This examination procedure complies with the current standards described in the latest medical literature [20]. With the use of the included software (digipaX) and a scaled radiograph, some measurements were taken: the tuberoarticular angle (Böhler angle), calcaneal pitch, also the surface shape (morphology) of the calcaneal tuberosity was assessed. On Figure 2 and Figure 3 the flat morphology of the calcaneal tuberosity is presented, seen in x-ray and ultrasonography. Additionally, on Figure 4 the bursa size measurement is shown.

The evaluation of the soft tissue surrounding the calcaneal tuberosity was made based on an ultrasonographic examination, with the use of the head at a frequency of 18 MHZ. The measurement was documented in two forms: a paper one and a digital one on a DVD. Every patient received their examination results thoroughly described and with a picture. The evaluated parameters were as following: the length of the Achilles tendon insertion, surface size of the bursa, the calcaneal tendon thickness, and the mobility of the bursa when the foot was actively moved by the patient.

### 2.3. Statistical Analysis

The studied group was approximately homogeneous in terms of other, unmeasured parameters. It was determined on the basis of interviews with the respondents that the studied group of people was approximately homogeneous due to other parameters. Thus, the statistically significant relationships found between the variable defining whether a certain person suffered from the disease and other variables indicate the existence of a real causal relationship between these variables, as there are no so-called hidden variables that could significantly affect the conclusions drawn. There exists a possibility that the values of certain variables, established during the research might have changed when compared with the period before the disease occurred. The analysis’s main aim was to make a general linear model (GLM), which would enable the risk of disease to be analyzed, depending on the parameters studied.

In the first stage of the analysis the correlation analysis was made in contingency tables, using an odds ratio of the disease, depending on the chosen characteristic’s value. Because the variable which was chosen to be analyzed comes from the binomial distribution, the logistic regression model was used at this point. In the next step, a Wald significance test was run, on the basis of which a logit model was suggested for the retrocalcaneal bursitis risk analysis.

All the calculations and analyses presented were made with the use of an R package (version 3.2.2., R Core Team, Vienna, Austria). The function GLM (Generalized Linear Models) from the stats package was used in order to fit the logistic regression model and to calculate the statistics connected with this process. The odds ratio and its asymptotic confidence intervals were calculated with the use of the author’s function. The basic statistics were also calculated using some functions from base and stats packages. The significance level was established to be α = 0.05 for two-sided tests.

## 3. Results

The analysis included 60 people (23 (38.3%) were women, 37 (62.7%) were men who met the criteria for inclusion in the study. The average age was 32 in the control group (SD = 7) and 35 in the test group (SD = 12). The average body mass index (BMI) was 22.7 kg/m^2^ (SD = 2.28) in the control group and in the test group—22.9 kg/m^2^ (SD = 3.11). In the control group the calcaneal tuberosity had flat shape in 16.7% people (*N* = 5) and in the tested group in 56.7% (*N* = 17) cases. In the test group the bursa was immobile in 83% (*N* = 25) of the feet that had suffered from inflammation, but also in 52% (*N* = 31) of the feet that had not had this inflammation. In Table 1 the descriptive statistics of chosen variables are presented.

Basic distribution of the variables (average, standard deviation, maximum, minimum, and median) carried out on the basis of x-ray imaging indicates that the talocalcaneal factor shows a very slight change in the control group (X = 1.3; SD = 0.1 for the left side and X = 1.3; SD = 0.1 for the right side) and in the test group (X = 1.3; SD = 0.1 for the left side and X = 1.4; SD = 0.1 for the right one). Ultrasound examination has shown a longer Achilles tendon insertion in the test group (X = 20; SD = 4.4 for the left and X = 19.4; SD = 3.9 for the right side) compared to the control group (SD = 18.9; SD = 3.6 for the left side and X = 17.3; SD = 2.2 for the right side). Similarly, in the test group a thicker Achilles tendon has been observed (X = 5.1; SD = 0.9 for the left side and X = 5.0; SD = 0.8 for the right side). Average dependence between the Achilles tendon thickness and the bursa surface has been shown, both in the control group (r = 0.56 for the left side and 0.57 for the right side) and in the test group (r = 0.57 for the left side and 0.59 for the right side). The correlations are all positive—when one parameter increases, the other one increases as well.

**Figure 5:**
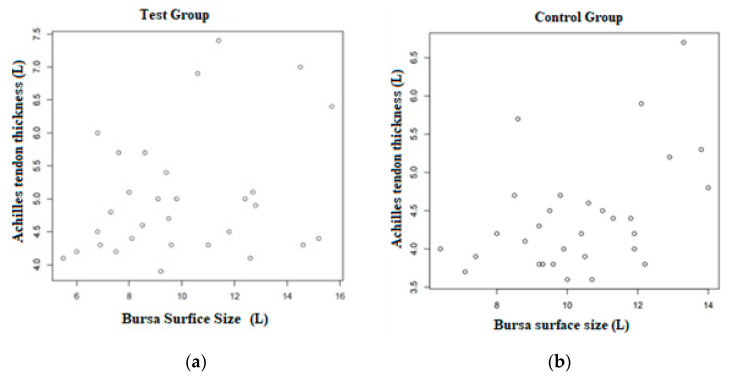
(**a**). A scatter graph presenting the correlation between the Achilles tendon thickness and the surface size of the bursa in the test group (*N* = 30). (**b**). A scatter graph presenting the correlation between the Achilles tendon thickness and the surface size of the bursa in the control group (*N* = 30).

The dependence of the Achilles tendon thickness on the surface size of the bursa has been the strongest correlation observed. In order to present this dependence better, it was shown in the scatter graphs below. Figure 5a presents parameters in the test group and Figure 5b in the control group.

The analysis of the likelihood of occurrence of retrocalcaneal bursitis on the basis of the odds ratio has been created using pre-prepared research questions. The results are presented in Table 2. In Table 3 more complex values are presented, along with their standard deviation, Wald test statistic values, and *p*-values for this test. The Wald test hypothesis is the irrelevance of the variable of the explanatory variable. Small *p*-values support rejection of this hypothesis.

The logit model suggested for the retrocalcaneal bursitis risk analysis is:

logit(p)=−6.546+1.348×(Achilles tendon thickness)+1.462×(calcaneal tuberosity shape)

−0.362×(bursa surface size).

The variable concerning the calcaneal tuberosity’s surface shape has a value of 1 when the tuberosity is flat and a value of 0 when it is normal, whereas *p* means probability of getting ill.

Particular attention should be paid to the possibility that parameters $e^{\beta }$, where β is the coefficient for an independent variable, might be interpreted using the odds ratio. In accordance with the values of structural parameters after estimation of the model concerned, it appears that the Achilles tendon 2 mm thicker than normal leads to a 15 times greater risk of the disease. On the other hand, when the bursa surface is 2 mm smaller than normal, the disease’s risk is more or less doubled.

## 4. Discussion

The aim of the study was to establish the possible relationship between the shape of the calcaneal tuberosity and the probability that retrocalcaneal bursitis should occur in people who train in running. The most important discovery is the fact that a flat surface shape of the calcaneal tuberosity and surface of the bursa being smaller than normal may increase the risk of inflammation in the area discussed.

There are few articles on the influence of the surrounding soft tissues on the Achilles tendon bursitis structure in literature. Bottger et al. from the University of Chicago [21] examined the MR imaging criteria for normal and abnormal retrocalcaneal bursae. Asymptomatic volunteers had average bursal dimensions of 1 mm in the anteroposterior dimension, 6 mm in the transverse dimension, and 3 mm in the craniocaudal dimension. Bursal dimensions greater than 1 mm, 11 mm, or 7 mm, respectively, were not seen in asymptomatic subjects but were seen in 16 (53%) of 30 ankles of patients with Achilles tendon disorders.

The examination of the calcaneus bone shape and its spatial orientation is very popular in up-to-date scientific studies. The researchers disagree on the fact that a prominent bone shape is related to the occurrence of inflammation in the distal insertion of the Achilles tendon. In 1982 Pavlov et al. [22] described the Haglund syndrome as the main cause of heel pain, which is characterized by soft tissue swelling in the calcaneal tendon indentation. On the other hand, a group of American scientists were the first to consider a disorder in the area of retrocalcaneal bursitis to be a possible result of the calcaneal tuberosity’s surface shape [23]. In later years, Kang et al. [24] examined a population of a hundred people, living in Los Angeles. According to this research, the calcaneal tuberosity’s surface shape was not related to any problems with the Achilles tendon. In this way, they questioned surgical treatment of this disease as the gold standard of treatment [25].

Suggested pathomechanism in case of flat calcaneal bone could be different. Anatomically, more horizontal calcaneus theoretically leaves greater surface for Achilles tendon attachment and smaller space for retrocalcaneal bursa. Additional repetitive trauma or stress overloading may change fluid density of bursa and cause inflammation. One of the arguments that stands for this theory is that 82% of people with flat surface of calcaneal spur had limited mobility of retrocalcaneal bursa in asymptomatic group of patients. All structures of the rear foot make up a single biomechanical system. Changes in one part of the system lead to adaptive changes in others and the whole system.

The study’s innovative approach was aimed at determining whether the flat surface shape of the calcaneal tuberosity may in an equally negative way influence retrocalcaneal bursitis. Similar reports in literature have not been found, so the problem seems to be remarkably interesting. In the test group, 57% of the people had flat calcaneal tuberosity, and in the control group—27%. Additionally, a flat bone shape is related to a smaller bursa surface and a thicker Achilles tendon. The data showed the relationship of the statistical significance of *p* < 0.001 for Achilles tendon thickness and *p* < 0.05 for bursa surface. On the basis of the modelling conducted, the conclusion can be drawn that when the bursa surface is only 2 mm smaller than normal, the risk of the retrocalcaneal bursitis is doubled. Therefore, it seems justified to treat all the strong associations collectively: calcaneal tuberosity shape—bursa surface—Achilles tendon thickness. The authors of scientific studies on imaging hindfoot structures stress a strong connection between these structures [26]. They also emphasize that this fact can be vital in the case of surgical treatment.

The conclusions drawn from this research project are close to Cohen’s approach [27]. He puts strong emphasis on the complexity of the problems that may appear in the hindfoot compartment. In his study, one can read about the perfect set created by soft tissues, together with the ankle’s tarsus and bones. This is in line with the study results discussed here as it describes the relationship between the anatomical surface shape of the calcaneal tuberosity and the soft tissue, that is the bursa. To our knowledge, this is the first study discussing the influence of the bone surface, the soft tissues and biomechanical variables. However, attention should be paid to the word ”optimal”, as it means such a combination of these variables that would be biomechanically the most proper for the person concerned.

In runners, reduction in mileage and avoiding workouts on hills can be helpful [28]. The use of heel lifts or oral anti-inflammatory agents is advised. The use of corticosteroid injection is controversial and brings about the risk of tendon rupture. Physiotherapy, particularly with stretching (in the purpose of minimizing frictions in the hindfoot) and eccentric-loading exercises, is recommended [29]. Stretching of the gastrocnemius-soleus complex is crucial to avoid Achilles contracture and excessive pressure on the calcaneal process [30].

## 5. Limitation of the Study

The study presented above has a few limitations. The biggest one seems to be a small number of people training running, which might have influenced the results. The expected effect size was derived from theoretical predictions. In order to confirm the dependencies discovered, a bigger number of runners should be evaluated. Additionally, doubts may arise whether the control and the test group were sufficiently similar to one another. In the sports discipline, such as running, many factors are important.

What is more, the study enables only to identify trends and assess the risk of retrocalcaneal bursitis. The other possible explanation is that retrocalcaneal bursitis may lead to thickening of the Achilles tendon. That should also be underlined. Therefore, no clear conclusions on the reciprocal impact of the variables examined can be drawn. The examinations took place at different times after the inflammation occurred, which may also influence the results. Furthermore, the ultrasonographic and x-ray controls were not carried out at the same time. To make sure that the results are simultaneously consistent, it would be best to consider a computed tomography examination or magnetic resonance imaging.

## 6. Conclusions

In conclusion, our study shows that there is a possibility that a flat surface shape of the calcaneal tuberosity may increase the risk of retrocalcaneal bursitis in people running. This inflammation’s risk can be higher when the Achilles tendon is thicker and surface of the bursa is smaller than normal. Therefore, it seems necessary to adjust every training plan individually, depending on one’s anatomical loads.

## Figures and Tables

**Table 1:** The descriptive statistics of chosen variables broken down by the control and test group.

Variable	Group	Average	SD	Min.	Max.	Median
Sex	I	W: 13 (43%)—M: 17 (57%)				
	II	W: 10 (33%)—M: 20 (67%)				
Hours of training weekly	I	6.9	3.3	4	18	6
	II	9.6	4.6	4	20	10
Kilometres run weekly	I	28.8	20.8	8	100	20
	II	38.9	29.4	10	120	35
Age [years]	I	32	7	19	56	30
	II	35	12	18	67	35
Weight [kg]	I	70.3	11.2	54	98	69
	II	70.5	13.6	48	108	71
Height [m]	I	1.75	0.1	1.6	1.8	1.7
	II	1.75	0.1	1.6	1.9	1.7

**Table 2:** The likelihood of occurrence of retrocalcaneal bursitis, depending on the value of the selected feature.

Variable	Or	95% CI
BMI > 25 kg/m^2^	2.25	(0.66; 7.69)
CP > 25^o^	2.8	(0.69; 11.32)
Insertion length < 13 mm	3.22	(0.51; 20.42)
Tendon thickness > 5 mm	4.38	(1.63; 11.75)
Flat calcaneal tuberosity	4.3	(1.68; 10.97)
Bursa surface < 9 mm	3.16	(1.23; 8.13)

List of abbreviations: CP—Calcaneal Pitch, OR—Odds Ratio, 95% CI—95% confidence interval, BMI—Body Mass Index.

**Table 3:** Assessment of significance of variables selected for the model based on Wald test.

Variable	Coefficient	SD	Wald Test	*p*-Vaule
Absolute term	−6.545	2.440	−2.682	0.007
Shape of calcaneal tuberosity	1.462	0.554	2.637	0.008
Bursa surface	−0.361	0.138	−2.608	0.009
Achilles tendon thickness	1.348	0.404	3.336	0.001

## References

[1] Poucher Z.A., Tamminen K.A., Caron J.G., Sweet S.N. (2020). Thinking through and designing qualitative research studies: A focused mapping review of 30 years of qualitative research in sport psychology. Int. Rev. Sport Exerc. Psychol..

[2] Fokkema T., Burggraaff R., Hartgens F., Kluitenberg B., Verhagen E., Backx F.J., Van Der Worp H., Bierma-Zeinstra S.M., Koes B.W., Van Middelkoop M. (2019). Prognosis and prognostic factors of running-related injuries in novice runners: A prospective cohort study. J. Sci. Med. Sport.

[3] Emery C.A., Pasanen K. (2019). Current trends in sport injury prevention. Best Pr. Res. Clin. Rheumatol..

[4] Van Der Worp M.P., Haaf D.S.M.T., Van Cingel R., De Wijer A., Der Sanden M.W.G.N.-V., Staal J.B. (2015). Injuries in Runners; A Systematic Review on Risk Factors and Sex Differences. PLoS ONE.

[5] Tiessen-Raaphorst A. (2015). Rapportage Sport 2014.

[6] Ooms L., Veenhof C., De Bakker D.H. (2013). Effectiveness of Start to Run, a 6-week training program for novice runners, on increasing health-enhancing physical activity: A controlled study. BMC Public Health.

[7] Ceyssens L., Vanelderen R., Barton C., Malliaras P., Dingenen B. (2019). Biomechanical Risk Factors Associated with Running-Related Injuries: A Systematic Review. Sports Med..

[8] Bertelsen M.L., Hulme A., Petersen J., Brund R.K., Sørensen H., Finch C.F., Parner E.T., Nielsen R.O. (2017). A framework for the etiology of running-related injuries. Scand. J. Med. Sci. Sports.

[9] Videbæk S., Bueno A.M., Nielsen R.O., Rasmussen S. (2015). Incidence of Running-Related Injuries Per 1000 h of running in Different Types of Runners: A Systematic Review and Meta-Analysis. Sports Med..

[10] Messier S.P., Martin D.F., Mihalko S.L., Ip E., DeVita P., Cannon D.W., Love M., Beringer D., Saldana S., Fellin R.E. (2018). A 2-Year Prospective Cohort Study of Overuse Running Injuries: The Runners and Injury Longitudinal Study (TRAILS). Am. J. Sports Med..

[11] Timpka T., Alonso J.-M., Jacobsson J., Junge A., Branco P., Clarsen B., Kowalski J., Mountjoy M., Nilsson S., Pluim B. (2014). Injury and illness definitions and data collection procedures for use in epidemiological studies in Athletics (track and field): Consensus statement. Br. J. Sports Med..

[12] Kisser R., Walters A., Rogmans W., Turner S., Lyons R.A. Injuries in the European Union. Summary of Injury Statistics for the Years 2013–2015. https://www.eurosafe.eu.com/uploads/inline-files/IDB%202013-2015_suppl%20to%206th%20edition%20Injuries%20in%20the%20EU.pdf.

[13] Doral M.N., Alam M., Bozkurt M., Turhan E., Atay O.A., Dönmez G., Maffulli N. (2010). Functional anatomy of the Achilles tendon. Knee Surg. Sports Traumatol. Arthrosc..

[14] Egger A.C., Berkowitz M.J. (2017). Achilles tendon injuries. Curr. Rev. Musculoskelet. Med..

[15] Hullfish T.J., Hagan K.L., Casey E., Baxter J.R. (2018). Achilles Tendon Structure Differs Between Runners and Non-Runners Despite No Clinical Signs or Symptoms of Mid-Substance Tendinopathy. BioRxiv.

[16] Lieberthal K., Paterson K.L., Cook J., Kiss Z., Girdwood M., Bradshaw E.J. (2019). Prevalence and factors associated with asymptomatic Achilles tendon pathology in male distance runners. Phys. Ther. Sport.

[17] Pierre-Jerome C., Moncayo V., Terk M.R. (2010). MRI of the achilles tendon: A comprehensive review of the anatomy, biomechanics, and imaging of overuse tendinopathies. Acta Radiol..

[18] Sundararajan P.P., Wilde T.S. (2014). Radiographic, Clinical, and Magnetic Resonance Imaging Analysis of Insertional Achilles Tendinopathy. J. Foot Ankle Surg..

[19] Deschamps K., Birch I., Desloovere K., Matricali G.A. (2010). The impact of hallux valgus on foot kinematics: A cross-sectional, comparative study. Gait Posture.

[20] Khurana A., Kadamabande S., James S., Tanaka H., Hariharan K. (2011). Weil osteotomy: Assessment of medium term results and predictive factors in recurrent metatarsalgia. Foot Ankle Surg..

[21] Bottger B.A., Schweitzer M.E., El-Noueam K.I., Desai M. (1998). MR imaging of the normal and abnormal retrocalcaneal bursae. Am. J. Roentgenol..

[22] Pavlov H., Heneghan M.A., Hersh A., Goldman A.B., Vigorita V. (1982). The Haglund syndrome: Initial and differential diagnosis. Radiology.

[23] Vega, Cavolo D., Green R., Cohen R. (1984). Haglund’s deformity. J. Am. Podiatr. Med. Assoc..

[24] Kang S., Thordarson D.B., Charlton T.P. (2012). Insertional Achilles Tendinitis and Haglund’s Deformity. Foot Ankle Int..

[25] Almaawi A., Boszczyk A.M., Daniels T.R. (2016). Exostosis (Osteochondrosis, Apophysites, and Haglund’s deformity). Foot and Ankle Sports Orthopaedics.

[26] Mahan J., Damodar D., Trapana E., Barnhill S., Nuno A.U., Smyth N.A., Aiyer A., Jose J. (2020). Achilles tendon complex: The anatomy of its insertional footprint on the calcaneus and clinical implications. J. Orthop..

[27] Cohen J.C. (2009). Anatomy and Biomechanical Aspects of the Gastrocsoleus Complex. Foot Ankle Clin..

[28] Damsted C., Glad S., Nielsen R.O., Sørensen H., Malisoux L. (2018). Is there evidence for an association between changes in training load and running-related injuries? A systematic review. Int. J. Sports Phys. Ther..

[29] Hulme A., Nielsen R.O., Timpka T., Verhagen E., Finch C. (2017). Risk and Protective Factors for Middle- and Long-Distance Running-Related Injury. Sports Med..

[30] Martin R.L., Chimenti R., Cuddeford T., Houck J., Matheson J., McDonough C.M., Paulseth S., Wukich D.K., Carcia C.R. (2018). Achilles pain, stiffness, and muscle power deficits: Midportion Achilles tendinopathy revision 2018: Clinical practice guidelines linked to the International Classification of Functioning, Disability and Health from the Orthopaedic Section of the American Physical Therapy Association. J. Orthop. Sports Phys. Ther..

